# Supplementary material for: Quantifying the effect of temporal resolution on time-varying networks
Source: Sci Rep. 2013 Oct 21;3:3006. doi: 10.1038/srep03006 (PMC3801130; doi:10.1038/srep03006)
Supplement: Supplementary Information — Quantifying the effect of temporal resolution on time-varying networks [file srep03006-s1.pdf]

Supplementary Information
Quantifying the effect of temporal resolution on time-varying networks

B. Ribeiro, N. Perra, A. Baronchelli

August 31, 2013

In this supplementary information appendix we cover some details left out of our main paper.

- Section 1 proves that a RW on an activity driven network is stationary and ergodic.
- Section 2 provides a detailed derivation of Eq.(1) in the main text.
- Section 3 analyzes the random walk occupancy probability when $\Delta t \to 0$.
- Section 4 analyzes the random walk occupancy probability in the special case of point-to-point time-varying networks $m = 1$.
- Section 5 analyzes the random walk occupancy probability on time-varying network of cliques in the $\Delta t \to 0$ scenario.
- And finally, Section 6 details our simulation results on real datasets.

1 RW stationarity and uniqueness conditions

An important requirement for the RW to be stationary and ergodic is for the network to be connected in time. A T-connected [5] time-varying network is a network that the aggregated network over $\Delta t \to \infty$ forms a connected graph (not necessarily fully connected). Consider some general stationary, ergodic, and T-conenected time-varying network with a fixed set of N nodes. From Theorem 3.1 of Figueiredo et al. [5] a RW on such network is stationary, and the stationary distribution is unique. To achieve this results we just need to translate the RW framework of Figueiredo et al. into our framework, which requires only setting parameter $\gamma \to \infty$ of the Figueiredo et al. RW, described in the paragraph after Definition 2.4).

2 Derivation of $Q_{a|a'}(\Delta t)$

Let $N \gg 1$ denote the total number of nodes in the graph. Let Ω be the set of all possible activity rates. There are no restrictions on the sample space Ω, which can be a discrete subset or a collection of continuous subsets. E.g., $\Omega = \{0.1, 0.2, 0.3\}$, another example is $\Omega = \{(0, 0.5), (0.8, 1)\}$, and our likely scenario $\Omega = (0, 1)$. Let $\mathrm{dF}(a)$ denote the probability that a randomly chosen node has

activity a. We write $\mathrm{dF}(a)$ instead of the more familiar density function $p(a)da$ because da may not be well defined if Ω is discontinuous or discrete. Let $V(t)$ be the node that the RW is at time $t\Delta t$ and let $A(t)$ denote the activity of node $V(t)$. If $A(t) = a$ then the number of times $V(t)$ is active during interval Δt, denoted $K_{\Delta t,a}$, is Poisson distributed in an activity driven network, i.e.,

$$\mathrm{P}[K_{\Delta t,a} = k] = \frac{(a\Delta t)^k}{k!}\exp(-a\Delta t).$$

Let $H_{\Delta t,a}$ be the number of times any other node in the network connects to $V(t)$ then

$$\mathrm{P}[H_{\Delta t,a} = h] \approx \frac{(m\langle a\rangle\Delta t)^h}{h!}\exp(-m\langle a\rangle\Delta t),$$

where above we use the fact that $N \gg 1$ so that $m(N\langle a\rangle - a)/(N-1) \approx m\langle a\rangle$. Thus, for all $a, a' \in \Omega$,

$$\begin{aligned}
&\mathrm{dP}[A((n+1)\Delta t) = a \,|\, A(n\Delta t) = a'] = \\
&= \sum_{k=0}^{\infty}\sum_{h=0}^{\infty} \mathrm{dP}[A((n+1)\Delta t) = a \,|\, A(n\Delta t) = a',\, K_{\Delta t,A(n\Delta t)} = k,\, H_{\Delta t,A(n\Delta t)} = h] \\
&\quad \times \mathrm{P}[K_{\Delta t,A(n\Delta t)} = k\,,\, H_{\Delta t,A(n\Delta t)} = h \,|\, A(n\Delta t) = a'] \\
&= \sum_{k=0}^{\infty}\sum_{h=0}^{\infty}\left(\frac{U_m(N;k)}{U_m(N;k)+h+\epsilon}\mathrm{dF}(a) + \frac{h}{U_m(N;k)+h+\epsilon}\frac{a\,\mathrm{dF}(a)}{\langle a\rangle} + \frac{\epsilon}{U_m(N;k)+h+\epsilon}\delta(a-a')\right) \\
&\quad \times \mathrm{P}[K_{\Delta t,a'} = k]\mathrm{P}[H_{\Delta t,a'} = h],
\end{aligned}$$

where $\epsilon \to 0$ and $U_m(N;k)$ is a the number of blue nodes in the graph after the following node coloring process:

1. Start with a set of N nodes all colored white;
2. pick m randomly sampled nodes chosen without replacement and color them blue;
3. repeat step 2 exactly k times;
4. $U_m(N;k)$ is the total number of blue nodes in the set.

This problem is known as the coupon collector problem with batch selections. Note that Pólya's urn model is a different model. In Pólya's model when a node of a particular color is drawn, that node is put back along with a *new* node of the same color, i.e., the size of the graph increases at each round.

In the regime where the network is large enough in respect to Δt, $N \gg 1$, such that with high probability an active node does not randomly choose the same neighbor twice in an interval Δt – that is, a time-varying edge appears only once in an interval Δt –, or more formally $P[U(N;k) < mK_{\Delta t,a}] \approx 0$, $\forall a \in \Omega$, yields

$$\begin{aligned}
Q_{a|a'}(\Delta t) = \lim_{\epsilon\to 0}\textstyle\sum_{k=0}^{\infty}\sum_{h=0}^{\infty}&\left(\tfrac{m\,k}{m\,k+h+\epsilon}\mathrm{dF}(a) + \tfrac{h}{m\,k+h+\epsilon}\tfrac{a\,\mathrm{dF}(a)}{\langle a\rangle} + \tfrac{\epsilon}{m\,k+h+\epsilon}\delta(a-a')\right) \\
&\times \tfrac{(a'\Delta t)^k}{k!}\exp(-a'\Delta t) \times \tfrac{(m\langle a\rangle\Delta t)^h}{h!}\exp(-m\langle a\rangle\Delta t)\,.
\end{aligned} \quad (1)$$

Eq. (1) is also valid for N small if the aggregated network has weights representing the number of times the same edge appears during the interval Δt. In such weighted aggregated network the random walk chooses a neighbor with probability proportional to the neighbor's edge weight. We take an in-depth look at RWs on weighted aggregated networks in the special case $m = 1$ shown in Section 4.

3 Special Case 1: $\Delta t \to 0$

Assumption 1. We assume $N \gg 1$ large enough such that $\mathrm{P}[U_m(N,k) < mK_{\Delta t,a}] \approx 0$, $\forall a \in \Omega$.

Recall that we defined $Q_{a|a'} \equiv \mathrm{dP}[A((n+1)\Delta t) = a \,|\, A(n\Delta t) = a']$. For all $a, a' \in \Omega$, $n \geq 0$,

$$Q_{a|a'}(\Delta t) = \sum_{k=0}^{\infty}\sum_{h=0}^{\infty}\left(\frac{km}{km+h+\epsilon}\mathrm{dF}(a) + \frac{h}{km+h+\epsilon}\frac{a\,\mathrm{dF}(a)}{\langle a\rangle} + \frac{\epsilon}{km+h+\epsilon}\delta(a-a')\right) \times \mathrm{P}[K_{\Delta t,a'} = k]\mathrm{P}[H_{\Delta t,a'} = h]. \quad (2)$$

Definition 1: Define $o(x)$ as an undefined function of x such that $\left|\frac{o(x)}{x}\right| \to 0$ as $x \to 0$.

The probabilities the a node with activity a is active under Assumption 1 are:

- $\mathrm{P}[K_{\Delta t,a'} \geq 1, H_{\Delta t,a} \geq 1] = o(\Delta t)$,
- $\mathrm{P}[K_{\Delta t,a'} = 1, H_{\Delta t,a} = 0] = a'\Delta t + o(\Delta t)$,
- $\mathrm{P}[K_{\Delta t,a'} = 0, H_{\Delta t,a} = 1] = m\langle a\rangle\Delta t + o(\Delta t)$,
- $\mathrm{P}[K_{\Delta t,a'} = 0, H_{\Delta t,a} = 0] = 1 - (a' + m\langle a\rangle)\Delta t + o(\Delta t)$,

Substituting the above equalities into (2) yields

$$Q_{a|a'}(\Delta t) = (1 - (a' + m\langle a\rangle)\Delta t)\delta(a-a') + \mathrm{dF}(a)a'\Delta t + \frac{a\,\mathrm{dF}(a)}{\langle a\rangle}m\langle a\rangle\Delta t \quad (3)$$

RW stationary distribution

Define $\rho_a(n) \equiv \mathrm{dP}[A(n\Delta t) = a]/(N\,\mathrm{dF}(a))$ as the RW occupancy probability. Define $\mathrm{d}\rho_a(n+1) \equiv \rho_a(n+1) - \rho_a(n)$ as the increase in probability from time $n\Delta t$ to time $(n+1)\Delta t$ that the walker is in a node with activity a. The quantity $\mathrm{d}\rho_a(n+1)$ is the probability that a walker that was at a node with activity a' *and* moved to a node with activity a minus the probability that the walker was in a node with activity a *and* moved to a node with activity a', integrated over all $a' \in \Omega\backslash\{a\}$.

More formally,

$$\begin{aligned}
\mathrm{d}\rho_a(n+1) =& \frac{1}{N\mathrm{dF}(a)} \int_{a' \in \Omega \setminus \{a\}} \mathrm{dP}[A((n+1)\Delta t) = a,\, A(n\Delta t) = a'] \qquad (4)\\
& - \mathrm{dP}[A((n+1)\Delta t) = a',\, A(n\Delta t) = a] \\
=& \frac{1}{N\mathrm{dF}(a)} \int_{a' \in \Omega} \mathrm{dP}[A((n+1)\Delta t) = a \,|\, A(n\Delta t) = a'] \mathrm{dP}[A(n\Delta t) = a'] \\
& - \mathrm{dP}[A((n+1)\Delta t) = a' \,|\, A(n\Delta t) = a] \mathrm{dP}[A(n\Delta t) = a] \qquad (5)\\
=& \frac{1}{N\mathrm{dF}(a)} \int_{a' \in \Omega} \left(\mathrm{dF}(a) a' \Delta t + \frac{a\, \mathrm{dF}(a)}{\langle a \rangle} m \,\langle a \rangle\, \Delta t \right) \mathrm{dP}[A(n\Delta t) = a'] \\
& - \int_{a' \in \Omega} \left(\mathrm{dF}(a') a \Delta t + \frac{a'\, \mathrm{dF}(a')}{\langle a \rangle} m \,\langle a \rangle\, \Delta t \right) \mathrm{dP}[A(n\Delta t) = a] \\
=& \frac{1}{N\mathrm{dF}(a)} \Delta t \left(\int_{a' \in \Omega} \mathrm{dF}(a) a'\, \mathrm{dP}[A(n\Delta t) = a'] + \int_{a' \in \Omega} a\, \mathrm{dF}(a) m \mathrm{dP}[A(n\Delta t) = a'] \right.\\
& \left. - \int_{a' \in \Omega} \mathrm{dF}(a') a\, \mathrm{dP}[A(n\Delta t) = a] - \int_{a' \in \Omega} a'\, \mathrm{dF}(a') m\, \mathrm{dP}[A(n\Delta t) = a] \right),
\end{aligned}$$

where in (5) we use the fact that $\mathrm{dP}[A((n+1)\Delta t) = a,\, A(n\Delta t) = a] - \mathrm{dP}[A((n+1)\Delta t) = a,\, A(n\Delta t) = a] = 0$ to add $\{a\}$ to the integral. Thus,

$$\begin{aligned}
\frac{\mathrm{d}\rho_a(n+1)}{\Delta t} =& \mathrm{dF}(a) \int_{a' \in \Omega} a'\, \mathrm{dP}[A(n\Delta t) = a'] + a\, \mathrm{dF}(a) m \int_{a' \in \Omega} \mathrm{dP}[A(n\Delta t) = a'] \\
& - a\, \mathrm{dP}[A(n\Delta t) = a] \int_{a' \in \Omega} \mathrm{dF}(a') - \mathrm{dP}[A(n\Delta t) = a] \int_{a' \in \Omega} a'\, \mathrm{dF}(a') m. \qquad (6)
\end{aligned}$$

Using our definition of $\rho_a(n) \equiv \mathrm{dP}[A(n\Delta t) = a]/(N\mathrm{dF}(a))$, Eq. (6) yields

$$\begin{aligned}
\frac{N\mathrm{dF}(a)\mathrm{d}\rho_a(n+1)}{\Delta t} =& \mathrm{dF}(a) \int_{a' \in \Omega} a'\, N\mathrm{dF}(a') \rho_{a'}(n) + a\, \mathrm{dF}(a) m \int_{a' \in \Omega} N\mathrm{dF}(a') \rho_{a'}(n) \\
& - a\, \rho_a(n) N\mathrm{dF}(a) \int_{a' \in \Omega} \mathrm{dF}(a') - \rho_a(n) N\mathrm{dF}(a) \int_{a' \in \Omega} a'\, \mathrm{dF}(a') m.
\end{aligned}$$

Dividing both sides by $N\mathrm{dF}(a)$ yields

$$\begin{aligned}
\frac{\mathrm{d}\rho_a(n+1)}{\Delta t} =& \int_{a' \in \Omega} a'\, \mathrm{dF}(a') \rho_{a'}(n) + a\, m \int_{a' \in \Omega} \mathrm{dF}(a') \rho_{a'}(n) \\
& - a\, \rho_a(n) \int_{a' \in \Omega} \mathrm{dF}(a') - \rho_a(n) \int_{a' \in \Omega} a'\, \mathrm{dF}(a') m \\
=& \int_{a' \in \Omega} a'\, \mathrm{dF}(a') \rho_{a'}(n) + a\, m \int_{a' \in \Omega} \mathrm{dF}(a') \rho_{a'}(n) - \rho_a(n)(a + \langle a \rangle m). \qquad (7)
\end{aligned}$$

Because the RW is stationary and ergodic, as walker progresses, i.e., $n \gg 1$, $\rho_a(n)$ reaches a stationary distribution. More precisely,

$$\lim_{n \to \infty} \mathrm{d}\rho_a(n) = 0.$$

Substituting the above limit in (7) and defining the stationary occupancy probability $\rho_a \equiv \lim_{n\to\infty} \rho_a(n)$ we get the following flow balance equations

$$\int_{a'\in\Omega} a' \, \mathrm{dF}(a')\rho_{a'} + a\, m \int_{a'\in\Omega} \mathrm{dF}(a')\rho_{a'} = \rho_a(a - \langle a\rangle m).$$

Define $\langle \rho_a \rangle = \int_{a\in\Omega} a\, \rho_a \mathrm{dF}(a)$ and then we simplify the above to

$$\rho_a = \frac{\langle \rho_a\rangle + am}{a + \langle a\rangle m}. \tag{8}$$

To obtain $\langle \rho_a \rangle$ observe that

$$\begin{aligned}
\langle \rho_a\rangle &= \int_{a\in\Omega} a\rho_a \mathrm{dF}(a) = \int_{a\in\Omega} \frac{a\left(\langle \rho_a\rangle + am\right)}{a + \langle a\rangle m}\mathrm{dF}(a) \\
&= \langle \rho_a\rangle \int_{a\in\Omega} \frac{a}{a + \langle a\rangle m}\mathrm{dF}(a) + \int_{a\in\Omega} \frac{a^2 m}{a + \langle a\rangle m}\mathrm{dF}(a) \\
\langle \rho_a\rangle &= \frac{m\beta_2}{1-\beta_1},
\end{aligned}$$

where

$$\beta_i = \int_{a\in\Omega} \frac{a^i}{a + \langle a\rangle m}\mathrm{dF}(a).$$

We note in passing that Eq. (8) is exactly the result in Perra et al. [1].

4 Special Case 2: $m = 1$

Consider a Poisson process where edges arrive to node $V(t)$ with rate $a' + \langle a\rangle$ and let $R_{\Delta t}$ be the total number of edges attached to node $V(t)$ during time window $(t\Delta t, (t+1)\Delta t]$. Note that the network is assumed stationary and thus $R_{\Delta t}$ does not depend on t. Moreover, $R_{\Delta t}$ is Poisson distributed with rate $(a' + \langle a\rangle)$,

$$P[R_{\Delta t} = r] = \frac{((a' + \langle a\rangle)\Delta t)^r}{r!} e^{-(a'+\langle a\rangle)\Delta t}.$$

Note that $R_{\Delta t}$ does not depend on t as the network process is stationary.

Next we randomly assign edges one of two of the following **types**: an edge is of type **passive** with probability $a'/(a' + \langle a\rangle)$ and of type **active** with probability $\langle a\rangle/(a' + \langle a\rangle)$. From the infinite divisibility property of the Poisson distribution, the the number of *passive* and *active* edges are Poisson distributed with parameters $a'\Delta t$ and $\langle a\rangle\Delta t$, respectively.

Edge types & weighted aggregated networks. The above model does not describe a network but rather just **edge arrivals** and **type assignments** at a node. Fortunately, such description suffices in activity driven networks. This happens because at the next RW step, the network reconstructs itself, allowing us to treat the coupled RW and network dynamics as a simple renewal process. Interestingly, the above model already considers multiple appearances of the same edge as long as the aggregated network is represented as a weighted aggregated network.

Static network representations of time-varying networks can weighted or unweighted. In **weighted aggregated networks**, edges in $G_t(\Delta t)$, where $G_t(\Delta t)$ is the result of the union of all the edges generated in the interval $[t\Delta t, (t+1)\Delta t)$ (see Figure 1 of our main paper), have integer weights that represent the number of times the edge appears during interval $[t\Delta t, (t+1)\Delta t)$. In **unweighted aggregated networks**, edges are unweighted. An edge is present in $G_t(\Delta t)$ if it appears one or more times during interval $[t\Delta t, (t+1)\Delta t)$; otherwise the edge is not present. Throughout this work we consider unweighted aggregated networks. However, one of our main results, namely Section 4 result on the random walk occupancy probability on activity driven networks with $m = 1$ concurrent edge creations, can be readily applied to weighted network representations as well.

From the point of view of the walker, a weighted network with integer edge weights has an equivalent multigraph. A multigraph is a graph that allows multiple edges between nodes. The multigraph is constructed as follows: for each edge (u, v) with weight $w \in \mathbb{N}$ in the weighted graph add w edges (u, v) in the multigraph. A RW on a multigraph, just like a RW on a weighted graph, selects a destination endpoint with probability proportional to the number of edges to that destination (its weight in the weighted graph). In the regime where the probability that a node connects to the same edge twice is close to zero – e.g., $N \gg 1$ is large enough in respect to Δt – then the weighted graph is a simple 0-1 graph with high probability (and thus equivalent to an unweighted network). In what follows we assume that the network is a multigraph graph, which encompasses the in special scenario of 0-1 graphs.

Derivations. Recall that the walker randomly chooses one destination out of the $R_{\Delta t}$ edges in the multigraph. Because the type of the first edge – passive or active – is selected randomly, the random walk choice of edge is statistically equivalent to committing to always choose the first edge before knowing its type. We wish to remind the reader that $V(t)$ has activity rate a'. The probability that the first edge has a *passive* destination is $a'/(a' + \langle a\rangle)$ and the probability that it has an *active* destination is $\langle a\rangle/(a' + \langle a\rangle)$. The probability that $V(t)$ has no edge after a time window of size Δt is $\zeta_{a',\Delta t} = e^{-(a'+\langle a\rangle)\Delta t}$. Then, the probability that the walker moves from $V(t)$ to an active destination with activity a is $(1 - \zeta_{a',\Delta t})\langle a\rangle/(a' + \langle a\rangle) \times a\mathrm{dF}(a)/\langle a\rangle = (1 - \zeta_{a',\Delta t})a\mathrm{dF}(a)/(a' + \langle a\rangle)$. The probability that the walker moves from $V(t)$ to a passive destination with activity a is $(1 - \zeta_{a',\Delta t})a'/(a' + \langle a\rangle) \times \mathrm{dF}(a) = (1 - \zeta_{a',\Delta t})a'\mathrm{dF}(a)/(a' + \langle a\rangle)$. The probability that the walker stays in $V(t)$ is $\zeta_{a',\Delta t}$, which is the probability that there are no edges out of $V(t)$. Thus, summing all these factors we obtain the probability that the walker moves from a node with activity a' to a node with activity a:

$$Q_{a|a'}(\Delta t) = \left(\frac{a\mathrm{dF}(a)}{a' + \langle a\rangle} + \frac{a'\mathrm{dF}(a)}{a' + \langle a\rangle}\right)(1 - \zeta_{a',\Delta t}) + \delta(a' - a)\zeta_{a',\Delta t} \tag{9}$$

$$= \frac{a + a'}{a' + \langle a\rangle}\mathrm{dF}(a)(1 - \zeta_{a',\Delta t}) + \delta(a' - a)\zeta_{a',\Delta t}. \tag{10}$$

The occupation probabilities $\{\rho_a\}_{\forall a\in\Omega}$, are the unique solution to the fixed point set of Chapman-Kolmogorov equations

$$\rho_a = \frac{1}{\mathrm{dF}(a)} \int_{a'\in\Omega} Q_{a|a'}(\Delta t)\rho_{a'}\mathrm{dF}(a')\,, \quad \forall a \in \Omega\,. \tag{11}$$

5 RW occupancy probability on time-varying networks of cliques. The $\Delta t \to 0$ case.

As $\Delta t \to 0$ network nodes are either isolated or belong to a clique. For instance, in the co-citation network assume we measure the time that authors submit their work to the journal at time t. Authors cannot submit work simultaneously to the same journal – although authors may to submit multiple articles at short bursts so that they end up in the same journal volume. At time t an author is either isolated – when the author did not submit a paper at time t – or connected in a clique formed by the co-authors of the paper submitted at time t. We can then use Theorem 3.4 of Figueiredo et al. [5] which shows that a RW on any (stationary, ergodic, and T-connected) time-varying network whose snapshots are cliques has uniform occupancy probability, that is, $\rho_a = 1/N$.

6 Simulation on Real Datasets

We simulate a RW on top of the datasets as follows. First we build as a series of static graphs $G_t(\Delta t)$, $t = 0, 1, \ldots, \lfloor T/\Delta t \rfloor$ from the dataset, where T is the time of the last event in the dataset. Given the initial condition that the RW starts at a random node $\pi_0 = (1/N, \ldots, 1/N)$ we obtain the RW occupancy probability at time T by right multiplying π_0 by $\prod_{t=0}^{\lfloor T/\Delta t \rfloor} P_t$, where P_t is the RW transition probability matrix of $G_t(\Delta t)$.

To obtain the matching theoretical predictions we first obtain $dF(a)$ from the data. Let $\mathrm{F}(a)$ be the fraction of nodes with activity greater or equal than a. By definition $\lim_{\epsilon \to 0} \mathrm{dF}(a) = \mathrm{F}(a) - \mathrm{F}(a + \epsilon)$. Choosing $\epsilon = 10^{-2}$ yields good results with low computational burden. Figure S1 plots the empirical $\mathrm{F}(a)$ against a of the PRL author activity for different aggregation windows, $\Delta t \in \{$one day, ten days, two months, 6 months$\}$. And Figure S2 plots the empirical $\mathrm{F}(a)$ against a of the Yahoo! Music song activity for different aggregation windows, $\Delta t \in \{$one second, one minute, one hour, six hours, and one day$\}$. The theoretical results of Figures 5 and 6 in our main paper were obtained with $\mathrm{dF}(a)$ computed from the dataset snapshots with $\Delta t = 1$ second for Yahoo! and $\Delta t = 1$ day for PRL.

Note that in snapshots created by projecting a bipartite network (if A is the original bipartite matrix, then the projected network adjacency matrix can be either AA^T or $A^T A$). In such projected networks as we increase Δt by a factor of $\alpha > 1$, $\Delta t' = \alpha \Delta t$ the increase in activity may be greater or less than α. This is because while there is an α increase in the average number of links from the one side of the network to the other, the growth in the number of connections between agents in the projected network does not necessarily increase with α. In order to take this non-trivial projection effect into account, we rescale our Δt as to best fit the observed data.

To evaluate the impact of the empirical $\mathrm{dF}(a)$ for different choices of Δt in the results of Figure 4 of our main paper, we recompute Eq. (11) using the empirical $\mathrm{dF}(a)$ obtained from $\Delta t = 60$ (one minute) instead of the empirical $\mathrm{dF}(a)$ obtained from $\Delta t = 1$ (second) as in the original figure. Figure S3 shows our results. We note that the main difference between the results obtained in Figure S3 and the ones in Figure 4 of our main paper are concentrated on low activity nodes, which are better modeled by the empirical $\mathrm{dF}(a)$ from $\Delta t = 1$. Comparing again the figures for high activity nodes shows that our analytical results are robust to the choice of Δt when extracting the empirical $\mathrm{dF}(a)$. Our final observation is then that in our datasets choosing the lowest resultion of Δt to obtain the empirical $\mathrm{dF}(a)$ works best.

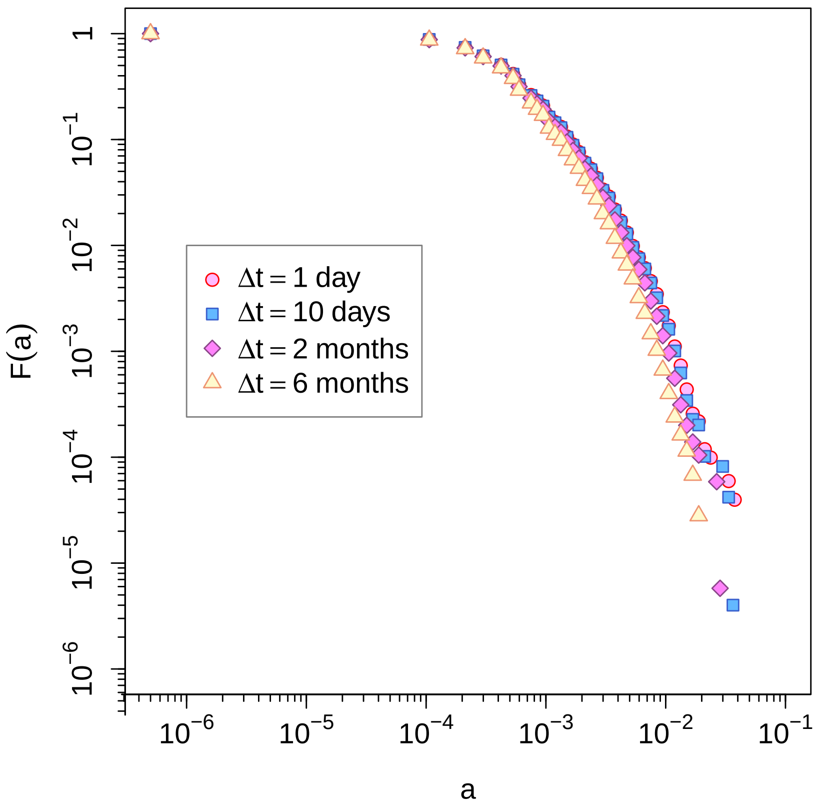

Figure S1: **(PRL dataset)** $F(a)$ for Δt of one day, ten days, two months, six months.

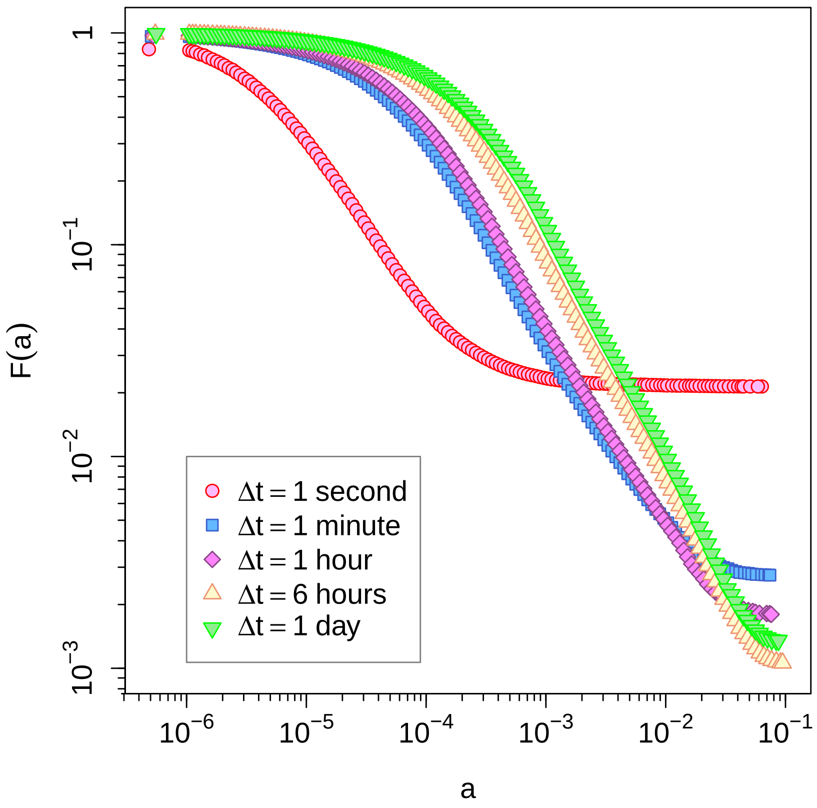

Figure S2: **(Yahoo! dataset)** $F(a)$ for Δt of one second, one minute, one hour, six hours, and one day.

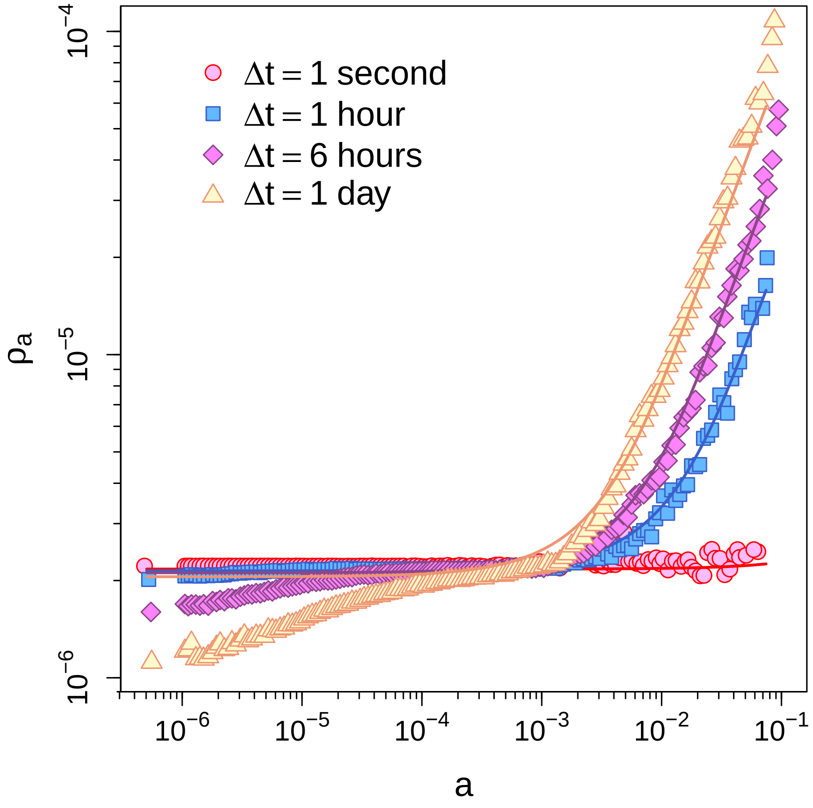

Figure S3: occupancy probability ρ_a of a RW at the end of the simulation as a function of node activity. This figure differs from Figure 4 of our main paper in that dF(a) in the main paper is obtained from Δt of one second and in the above figure dF(a) is obtained from Δt of 60 seconds. The points are the values of ρ_a on the time-varying graph of Yahoo! song ratings for different integrating windows Δt of one second, one hour, six hours, and one day. The solid lines are the numerical solution of Eq. (11). The errors bars are not visible in this case.

References

[1] N. Perra, B. Gonçalves, R. Pastor-Satorras, and A. Vespignani. Time scales and dynamical processes in activity driven networks. *Scientific Reports*, 2:469, 2012.

[2] APS. Data sets for research, 2010.

[3] Yahoo! webscope dataset, 2012.

[4] N. Perra, A. Baronchelli, D Mocanu, B. Gonçalves, R. Pastor-Satorras, and A. Vespignani. Random walks and search in time varying networks. *Phys. Rev. Lett.*, 109:238701, 2012.

[5] D Figueiredo, P Nain, B Ribeiro, E. de Souza, and D. Towsley. Characterizing continuous time random walks on time varying graphs. *SIGMETRICS*, pages 203–207, 2012.
